# Pharmacogenomics and Opioid Efficacy in Sickle Cell Disease: Is the Field Ready for Precision Prescribing?

**DOI:** 10.3390/jpm16080424

**Published:** 2026-08-11

**Authors:** Cheedy Jaja, Daniel M. Sop, Andrew Campbell, Wally R. Smith

**Affiliations:** 1 Department of Psychiatry, Virginia Commonwealth University, Richmond, VA 23219, USA; cheedy.jaja@vcuhealth.org; 2 Department of Internal Medicine, Virginia Commonwealth University, Richmond, VA 23298, USA; daniel.sop@vcuhealth.org; 3 Children’s National Hospital, Washington, DC 20010, USA; acampbell@childrensnational.org

**Keywords:** sickle cell disease, pharmacogenomics, CYP2D6, opioid prescribing, pain management, precision medicine, codeine, tramadol

## Abstract

Pain is a leading cause of morbidity and healthcare utilization in sickle cell disease (SCD), and opioids remain central to treating vaso-occlusive and chronic pain. Yet opioid response varies widely, raising the question of whether pharmacogenetic testing should inform opioid prescribing. Our review examined the PubMed literature on pharmacogenomics and opioid efficacy in SCD. We focus on CYP2D6 as the clearest current pharmacogenetic signal for codeine and tramadol, and assess SCD-specific implementation studies, preemptive testing, and African pharmacoequity. The current evidence supports targeted CYP2D6-informed prescribing in selected contexts rather than universal testing for all opioids, while highlighting the need to integrate genotype with pain phenotype, drug–drug interactions, liver function, and clinically grounded implementation studies, and better characterize African and African-ancestry pharmacogene variation.

## 1. Introduction

Sickle cell disease (SCD) is one of the most common inherited disorders worldwide and remains a major cause of morbidity because of recurrent pain, organ injury, and repeated healthcare utilization [1,2]. Pain is its most frequent and disabling manifestation. It includes acute vaso-occlusive episodes, acute-on-chronic pain, and chronic pain syndromes that may be managed at home or in emergency and inpatient settings. Recurrent exposure to analgesics begins early in life and continues across adulthood. This combination of severe pain, repeated treatment, and variable clinical context makes individualized prescribing especially important in SCD [1,2].

Opioid response in SCD is heterogeneous. Some patients have inadequate analgesia despite apparently appropriate dosing, whereas others develop oversedation, toxicity, or dose-limiting adverse effects [3,4,5]. These differences reflect more than pain intensity. Disease biology, organ dysfunction, chronic inflammation, pain mechanism, concomitant medications, and inherited variation in drug metabolism can all influence response [1,3,4,5]. Trial-and-error prescribing is therefore particularly problematic in SCD, where delayed pain control may prolong suffering, increase acute-care use, and deepen mistrust between patients and clinicians.

Pain in SCD is also heterogeneous in mechanism and trajectory. Acute vaso-occlusive pain may coexist with chronic nociceptive, neuropathic, or nociplastic features, and the same patient may transition among these states [1,2]. Average pain intensity alone may not capture day-to-day variability, functional restriction, sleep disruption, or psychosocial burden. Precision prescribing must therefore address both the pharmacology of the selected drug and the clinical phenotype of the pain being treated. Pharmacogenomics can inform the former, but it cannot replace a multidimensional pain assessment.

Pharmacogenomics offers one route to more precise prescribing. Among candidate pharmacogenes, CYP2D6 has the clearest and most mature actionable guidance for codeine and tramadol, which require CYP2D6-mediated bioactivation to form their principal active metabolites [4,5,6]. Reduced CYP2D6 function can contribute to treatment failure, whereas ultrarapid metabolism can increase active-metabolite exposure and toxicity. Other genes, including UGT2B7, OPRM1, COMT, CYP2B6, and ABCB1, have been associated with opioid response, but their current prescribing implications are less consistent [3,4,5,6].

The opportunity extends beyond opioids. Patients with SCD receive multiple medications over time, many of which have established pharmacogenetic guidance. Examples include codeine and tramadol through CYP2D6, selected nonsteroidal anti-inflammatory drugs through CYP2C9, ondansetron through CYP2D6, proton pump inhibitors through CYP2C19, and selected antidepressants through CYP2D6 and CYP2C19 [7]. A reusable pharmacogenomic result could therefore inform several prescribing decisions across the lifespan. However, the clinical value of testing depends on whether a gene–drug relationship is sufficiently actionable and whether results can be interpreted within the patient’s current clinical context.

The practical question is not whether inherited variation can influence drug response, but when testing produces information that is actionable enough to change care. A clinically useful test should identify a meaningful risk of treatment failure or toxicity, provide a clear alternative, and be available when the prescribing decision is made. These criteria are met most convincingly for CYP2D6 and codeine or tramadol. They are less clearly met for other opioids or for broad multigene panels used without a defined clinical pathway.

The primary objective of this narrative review is to determine whether current evidence supports pharmacogenetically informed opioid prescribing in SCD, particularly before use of codeine or tramadol. Secondary objectives are to evaluate the relative actionability of CYP2D6 across commonly used opioids, synthesize SCD-specific implementation evidence, examine clinical factors that limit genotype-only prescribing, and identify priorities for research and equitable implementation. We distinguish what is ready for targeted clinical use from what is promising but still requires SCD-specific validation.

## 2. Review Methodology

This narrative review examined pharmacogenetic and pharmacogenomic evidence relevant to opioid prescribing and pain management in SCD. Literature searches were conducted in PubMed from January 2013 through May 2026 using combinations of the terms “sickle cell disease,” “pharmacogenomics,” “pharmacogenetics,” “CYP2D6,” “opioids,” “codeine,” “tramadol,” “pain,” “phenoconversion,” “implementation,” and “Africa.” We included English-language peer-reviewed studies, clinical guidelines, implementation reports, and reviews addressing opioid pharmacogenetics in SCD or evidence directly relevant to its clinical translation. SCD-specific studies and prescribing guidelines were prioritized. Non-SCD studies were included when they informed opioid gene–drug actionability, preemptive panel testing, or implementation models. Reference lists of relevant articles were also reviewed. Because this was a narrative review, no formal risk-of-bias assessment or meta-analysis was performed, and recently published studies were preferentially selected.

The review was organized around four questions: which opioid gene–drug pairs have actionable guidance; what evidence is specific to SCD; which clinical factors limit genotype-only prescribing; and what research and implementation steps are required for broader adoption. The search was intended to support a critical narrative synthesis rather than an exhaustive enumeration of every opioid pharmacogenetic association.

## 3. CYP2D6 and the Evolution of Actionable Opioid Pharmacogenetics

### 3.1. From Mechanistic Discovery to Clinical Guidance

Opioid pharmacogenetics has moved from broad mechanistic investigation toward selective prescribing guidance [8,9]. Early studies examined inherited variation in drug-metabolizing enzymes, opioid receptors, transporters, and downstream signaling pathways. Over time, the most clinically interpretable evidence converged on CYP2D6 and opioids that depend on CYP2D6-mediated bioactivation. The principal advance was not validation of every plausible opioid-related gene. It was identification of a smaller set of gene–drug pairs that can support concrete prescribing decisions [8,9].

The Clinical Pharmacogenetics Implementation Consortium (CPIC) guideline for CYP2D6, OPRM1, COMT, and selected opioids reflects this transition [8]. The guideline provides the strongest therapeutic recommendations for codeine and tramadol. It does not support routine genotype-based prescribing for OPRM1 or COMT, and the evidence is less definitive for hydrocodone and oxycodone [8]. This asymmetry is important. It argues against treating “opioid pharmacogenetics” as a single level of evidence and instead requires drug-specific interpretation.

Implementation has become the next challenge. Programs increasingly use preemptive genotyping, standardized genotype-to-phenotype translation, and electronic health record decision support [9,10]. At Cincinnati Children’s Hospital Medical Center, CYP2D6 testing began with codeine and evolved as assays, CPIC recommendations, regulatory guidance, and electronic reporting changed [10]. The program required collaboration among laboratories, pharmacists, clinicians, informatics specialists, and pain teams. Prescriber education combined with decision support reduced use of CYP2D6-dependent opioids in patients with high-risk phenotypes, although the study was not designed to establish clinical effectiveness [10]. The central lesson is that actionable evidence alone is insufficient; clinical benefit also depends on accurate results, accessible interpretation, and usable workflow integration.

Implementation experience also reframes pharmacogenetics as a safety-preserving strategy rather than a mechanism for restricting analgesic access. The goal is to avoid predictable nonresponse or toxicity while preserving effective options for patients whose phenotype supports standard use [5,8]. This distinction is especially important in SCD, where undertreatment and stigmatizing assumptions about opioid use remain major concerns. Pharmacogenetic guidance should narrow uncertainty around a specific drug, not justify generalized opioid avoidance.

### 3.2. CYP2D6 Phenotype Assignment and Activity Scores

CYP2D6 genotype is commonly translated into a predicted metabolic phenotype using an activity-score framework. Each allele is assigned a functional value, and the values are summed to estimate enzyme activity [8]. Under the CPIC framework used in this review, an activity score of 0 corresponds to a poor metabolizer, 0.25–1.0 to an intermediate metabolizer, 1.25–2.25 to a normal metabolizer, and greater than 2.25 to an ultrarapid metabolizer [8]. This graded framework is more clinically useful than a simple functional/nonfunctional distinction because active-metabolite formation changes across a continuum.

Table 1 summarizes the expected implications for codeine and tramadol. The phenotype is a prediction rather than a fixed measure of current enzyme activity. Concomitant CYP2D6 inhibitors can reduce actual activity and produce phenoconversion, a limitation considered in Section 7 below. Copy-number variation, structural variants, and incompletely characterized alleles may also complicate phenotype assignment, particularly in genetically diverse populations [8].

Phenotype assignment also depends on the quality and scope of the assay. Limited panels may miss rare or ancestry-enriched variants, and duplication calls may not identify which allele is duplicated. Laboratories should report both the diplotype and the translated phenotype, describe assay limitations, and update interpretation when consensus activity scores change [8,10]. These requirements are particularly relevant to preemptive testing, because a result may be used repeatedly over many years.

### 3.3. Codeine and Tramadol: The Strongest Actionable Pairs

Codeine and tramadol provide the clearest current use case for CYP2D6-guided opioid prescribing [5,8]. Codeine requires CYP2D6 conversion to morphine, while tramadol requires CYP2D6 conversion to O-desmethyltramadol for much of its opioid effect. Poor metabolizers may generate too little active metabolite and experience inadequate analgesia. Ultrarapid metabolizers may generate active metabolite quickly and at higher concentrations, increasing the risk of serious toxicity at standard doses [8].

CPIC recommends avoiding codeine and tramadol in poor metabolizers because of likely treatment failure and in ultrarapid metabolizers because of toxicity risk [8]. Intermediate metabolizers may receive a standard starting dose but should be monitored for inadequate response, with a non-CYP2D6-dependent alternative considered when pain control is insufficient. Normal metabolizers may receive standard label-recommended dosing [8]. These recommendations are supported by mechanistic evidence, replicated associations with metabolite exposure and clinical response, and formal prescribing guidance. Together, those features make codeine and tramadol the most defensible “ready now” targets for opioid pharmacogenetics.

The clinical value of testing is greatest when it changes the drug choice before exposure. For a poor or ultrarapid metabolizer, selecting an opioid that does not require CYP2D6 bioactivation avoids a predictable mechanism of failure or toxicity. For an intermediate metabolizer, the result may justify closer monitoring and earlier switching if analgesia is inadequate. A normal-metabolizer result does not guarantee good pain control, because pain mechanism, dose, adherence, organ function, and interacting medications remain important.

Evidence outside SCD supports this hierarchy. In oncology, intermediate and poor metabolizers receiving commonly used CYP2D6-dependent opioids had more pain-related encounters and were more likely to require later-line agents such as morphine or hydromorphone [11]. Randomized and pragmatic postoperative studies also suggest that genotype-guided strategies may improve opioid selection, although observed benefit depends on the drug prescribed and the surrounding multimodal analgesic regimen [12,13]. These studies support selective CYP2D6-guided prescribing rather than broad claims that all opioid response can be predicted genetically.

Even for codeine and tramadol, implementation should distinguish genotype prediction from observed response. A patient who has previously tolerated and benefited from a drug provides clinically relevant evidence, while a new prescription carries greater uncertainty. Testing is most useful when no reliable treatment history is available, when prior response is unexplained, or when the clinical setting requires repeated future prescribing. The result should supplement, not displace, prior medication history and bedside assessment.

### 3.4. Hydrocodone and Oxycodone: Biologically Relevant but Less Settled

Hydrocodone is converted by CYP2D6 to hydromorphone, a more potent metabolite, but the parent drug retains opioid activity. Reduced CYP2D6 activity therefore lowers hydromorphone formation without consistently predicting overall analgesic failure [8,13]. CPIC does not recommend routine avoidance or genotype-based dose adjustment. Instead, usual dosing is appropriate, with consideration of an alternative analgesic when an intermediate or poor metabolizer has an inadequate response [8]. Hydrocodone is thus clinically relevant but less actionable than codeine or tramadol.

This distinction has practical consequences for reporting. A laboratory report that lists hydrocodone alongside codeine and tramadol without grading the evidence may imply equivalent actionability. Reports and decision support should instead communicate that hydrocodone recommendations are conditional on inadequate response and that alternative selection remains a clinical judgment [8,10].

Oxycodone is converted by CYP2D6 to oxymorphone, but oxycodone itself has substantial activity. Studies have not consistently linked CYP2D6 phenotype with oxycodone consumption or pain intensity, especially in settings using multimodal analgesia [8,12,13]. CPIC therefore makes no genotype-based recommendation for oxycodone. Recent postoperative analyses similarly found no clear association between predicted phenotype and outcomes for oxycodone or hydrocodone [13]. Current evidence does not support routine CYP2D6-guided oxycodone prescribing.

Null or inconsistent findings may reflect the intrinsic activity of the parent drug, heterogeneity in dosing and co-analgesics, and differences between acute postoperative pain and recurrent SCD pain. They do not prove that CYP2D6 has no pharmacologic effect. Rather, they show that the effect is not sufficiently predictable to support a routine prescribing recommendation at present [12,13].

The practical conclusion is narrow but useful. CYP2D6 has the clearest actionable opioid guidance for codeine and tramadol. Hydrocodone may warrant additional consideration after inadequate response, while oxycodone remains biologically plausible but clinically unsettled [8,13]. This formulation is more defensible than describing CYP2D6 as the only relevant opioid pharmacogene or assuming that one genotype result applies equally to all opioids.

## 4. SCD-Specific Evidence and Implementation

### 4.1. Pediatric Implementation at St. Jude

Recent SCD-specific studies reduce reliance on evidence extrapolated from oncology, psychiatry, and perioperative care [5,14,15]. The St. Jude Children’s Research Hospital program provides a pediatric example of genotype-guided codeine prescribing in SCD [14]. CYP2D6 genotyping was incorporated into the electronic health record and linked to pretest and posttest clinical decision-support alerts. Among 830 active pediatric patients with SCD, 621 had a CYP2D6 result. Approximately 1.4% were poor metabolizers and 7% were ultrarapid or possible ultrarapid metabolizers. Excluding indeterminate results, the investigators estimated that 87% could receive label-recommended codeine doses, while 9% had high-risk results [14].

The program required a known CYP2D6 result before codeine prescribing. Alerts were triggered when no result was available or when a high-risk phenotype was present. Tramadol was not recommended as an alternative because it has similar CYP2D6 dependence. Intermediate metabolizers received advice to use another agent if analgesia was inadequate [14]. This strategy used pharmacogenetics to avoid predictable risk without eliminating codeine for patients likely to respond. No severe adverse events were reported among patients with SCD who received codeine in the analysis [14].

The program was developed in a context where codeine remained clinically useful and access to alternatives was changing. This is important because pharmacogenetics was used to preserve an established treatment option for most patients while protecting a smaller high-risk group [14]. The approach demonstrates that precision prescribing can support access as well as safety, provided that recommendations are drug-specific and embedded in routine care.

The St. Jude experience also illustrates the importance of implementation design. Testing was useful because results were available before prescribing, linked to decision support, and accompanied by recommendations. A genotype result stored without interpretation or workflow integration would have been less likely to change care. The study therefore supports targeted use of CYP2D6 in SCD while showing that safety depends on systems, not genotype alone.

However, the St. Jude report was an implementation study rather than a randomized comparison. It established feasibility, coverage, and safe prescribing behavior, but it did not determine whether genotype-guided codeine improved pain relief, reduced hospital utilization, or outperformed an alternative opioid strategy. Those outcome questions remain open.

### 4.2. The Nigerian iPROTECTA Cohort

The Nigerian iPROTECTA program provides the largest recent SCD-specific CYP2D6 implementation cohort [15]. Investigators genotyped 503 patients with SCD at five sites. Phenotype frequencies were 8.8% ultrarapid, 54.1% normal, 26.0% intermediate, 1.8% poor, and 9.3% undetermined metabolizers [15]. Actionable phenotypes were therefore common. Under CPIC recommendations, poor and ultrarapid metabolizers should avoid codeine and tramadol, while intermediate metabolizers require monitoring for inadequate analgesia.

The undetermined group is also clinically important. It reflects the limits of current genotype-to-phenotype translation and may include structural or population-specific variation not fully captured by the assay. A program must define how clinicians should prescribe when a result is uncertain, rather than treating “undetermined” as equivalent to normal metabolism.

The program paired genotype interpretation with a patient medication safety card and a physician-facing report containing treatment recommendations [15]. These tools were designed for clinical environments where a fully integrated electronic health record may not be available. The study also addressed limited clinician familiarity, laboratory capacity, and laboratory-clinic coordination through a Pan-African collaborative model. It therefore provides evidence not only about phenotype prevalence but also about feasible implementation in a resource-limited setting.

The Nigerian findings should not be interpreted as proof that preemptive testing improves pain outcomes. The study was an implementation phase rather than a definitive comparative-effectiveness trial. Nevertheless, it shows that a substantial proportion of patients may have a phenotype relevant to codeine or tramadol and that results can be communicated through locally practical tools [15]. The next step is to determine whether this approach reduces analgesic failure, toxicity, opioid escalation, or acute-care utilization.

Outcome evaluation should also examine how often clinicians use the result, whether patients present the safety card across institutions, and whether recommendations remain accessible during urgent pain encounters. These implementation endpoints are particularly important in SCD, where care is often fragmented across outpatient, emergency, and inpatient settings.

### 4.3. African and African-Ancestry CYP2D6 Variation

Other African and African-ancestry studies reinforce the need for population-specific evidence. In 75 African-American children with SCD, 44.0% were predicted intermediate metabolizers, 6.7% poor metabolizers, and 4.0% ultrarapid metabolizers; the reduced-function CYP2D6*17 allele had a frequency of 17.3% [16]. The study also identified genotype-phenotype discordance and indeterminate duplication calls, illustrating the difficulty of phenotype assignment when structural variation is incompletely resolved [16].

In Zimbabwean patients with SCD, CYP2D6*17 and *29 were common, and UGT2B7 variation was associated with pain levels [17]. A Ghanaian population analysis similarly reported intermediate and poor metabolizer phenotypes relevant to tramadol exposure [18]. These studies are not directly interchangeable because they differ in design, population, assay, and clinical endpoint. Collectively, however, they show that reduced-function alleles and complex phenotype translation are important in populations carrying a large proportion of the global SCD burden [16,17,18].

Assay design is a major source of heterogeneity. CYP2D6 includes deletions, duplications, hybrid genes, and alleles with reduced rather than absent function. The same nominal phenotype can therefore arise from different diplotypes, and limited panels may classify some results incorrectly or leave them unresolved. This complexity strengthens the case for ancestry-aware laboratory methods and transparent reporting of uncertainty [16,19].

Recent SCD genomics reviews argue that clinically valid precision medicine must include African cohorts because genomic diversity can affect allele frequency, structural variation, and treatment response [19]. SCD-specific pharmacogenetic guidance should therefore be tested in the populations where it will be used rather than inferred primarily from European-ancestry datasets. This is both an equity requirement and a condition of scientific validity [19].

At the same time, population-level frequencies should not be used as a substitute for individual testing. Ancestry can guide assay design and evidence generation, but it cannot reliably predict one patient’s genotype. Clinical implementation should avoid racialized prescribing shortcuts and base recommendations on validated individual results interpreted within the relevant population context.

## 5. Preemptive Pharmacogenomics Across the SCD Lifespan

The strongest argument for preemptive pharmacogenomics in SCD is cumulative medication exposure. A result obtained early could inform repeated prescriptions rather than a single opioid decision [7,20,21,22]. In an Indiana University cohort of 957 patients with SCD, 93% received at least one medication with actionable CPIC guidance over 16 years, with a mean of 3.32 such drugs per patient [7]. Opioids and nonsteroidal anti-inflammatory drugs were most common, followed by 5-hydroxytryptamine-3 receptor antagonists and proton pump inhibitors. Exposure began early and increased with age and duration of follow-up [7].

In that cohort, opioids were prescribed to 85.1% of patients and nonsteroidal anti-inflammatory drugs to 80.1%; 5-hydroxytryptamine-3 receptor antagonists and proton pump inhibitors were also common [7]. Median initiation occurred in adolescence for opioids and nonsteroidal anti-inflammatory drugs. The repeated opportunities for actionable prescribing distinguish SCD from a one-time treatment setting and provide the main rationale for obtaining results before an urgent need arises.

These data support a reusable multigene profile that could include CYP2D6, CYP2C19, and CYP2C9 [7]. Such a profile might inform opioids, selected nonsteroidal anti-inflammatory drugs, antidepressants, proton pump inhibitors, and antiemetics across the lifespan. However, the Indiana study measured prescribing opportunities rather than clinical benefit. It did not establish that preemptive testing improves analgesia, prevents toxicity, or reduces utilization in SCD.

A preemptive strategy also depends on durable storage and portability. Results obtained in childhood must remain available when patients transition to adult care, receive treatment at another institution, or present to an emergency department. Without reliable access and periodic reinterpretation, the theoretical value of a reusable result may not translate into clinical benefit.

Broader implementation studies provide proof of principle. Reviews of preemptive panel programs describe integration of multigene results into electronic health records and decision-support systems [21]. The PREPARE study, conducted across seven European healthcare systems, reported a 30% reduction in clinically relevant adverse drug reactions with a 12-gene panel and genotype-guided prescribing [21]. A Swiss 16-gene panel study found at least one actionable variant in every participant and generated recommendations for current and future medications [20]. These studies demonstrate feasibility and potential value but are not SCD-specific.

In the Swiss cohort, panel results led to recommendations to change the triggering medication in 32.4% of patients tested for a current drug indication and to adjust concomitant medication in 22.5% [20]. Expert review reduced numerous automated alerts to a smaller set of clinically prioritized recommendations. This illustrates why pharmacist or pharmacogenetics expertise may remain necessary even when reports are generated electronically.

These programs also show that the value of a panel is not simply the number of variants detected. Clinical utility depends on whether a future prescription activates a relevant recommendation and whether clinicians act on it. A panel that generates numerous low-priority alerts may increase complexity without improving care, whereas a smaller set of clearly actionable results may be more useful.

Preemptive testing also introduces complexity. In the Swiss cohort, CYP2D6 and CYP2C19 inhibitors and impaired renal function altered the clinical interpretation of genotype results [20]. Expert review was needed to prioritize automated alerts. In African settings, local allele frequencies, prescribing patterns, infrastructure, and disease burden may require a panel different from one designed for European populations [22].

The same cohort found that 19.3% of patients were taking a CYP2D6 inhibitor, 8.2% a CYP2C19 inhibitor, and 14.1% had impaired renal function [20]. These factors changed the practical interpretation of genetic results and reinforced the need for dynamic clinical review. A preemptive genotype may be stable, but the medication list and organ function are not.

Preemptive pharmacogenomics across the SCD lifespan is therefore promising but not yet standard care. Its rationale is stronger than the evidence for its SCD-specific outcomes. Prospective studies must establish whether early, reusable results improve medication selection, reduce adverse effects, and remain useful across transitions from pediatric to adult care.

## 6. African Pharmacoequity and Genomic Representation

Because much of the global burden of SCD lies in Africa, clinically valid precision prescribing depends on African representation, locally relevant implementation, and pharmacoequity [15,18,19,22,23,24]. Underrepresentation of African populations in genomic datasets can reduce the accuracy of allele interpretation and phenotype assignment. This is particularly important for CYP2D6, where copy-number variation, reduced-function alleles, and incompletely characterized haplotypes can materially affect opioid response [15,16,18,19].

African cohort data should therefore be treated as a validity issue rather than an optional diversity objective. South African analyses caution that guidelines and panels derived mainly from European or Asian populations may misclassify metabolizer status or prioritize variants with limited local relevance [22,24]. Regional genomic diversity, disease burden, and first-line treatment patterns should inform panel design and implementation. The African and African-ancestry findings summarized in Section 4 illustrate why a single ancestry-neutral evidence base may be insufficient.

Locally informed panels may need to include variants that are uncommon in European datasets but frequent or functionally important in African populations. They may also need different strategies for copy-number analysis and uncertain haplotypes. Validation should include analytic accuracy, phenotype translation, and prospective clinical outcomes rather than allele frequency alone [19,22,24].

Implementation must also fit local health systems. Recent African reviews identify barriers that include limited laboratory and sequencing capacity, fragmented funding, insufficient clinician education, weak regulatory frameworks, limited informatics, and concerns about data ownership and community trust [23,24]. These barriers are not peripheral. They determine whether a result can be generated accurately, interpreted, communicated, and acted upon.

Ethical and governance considerations are equally important. Community engagement, informed consent, data security, data ownership, and benefit sharing must be addressed in ways that fit local legal and cultural settings [23,24]. Low literacy, rural residence, and fragmented care may influence how results are communicated and retained. Implementation that overlooks these factors may produce technically accurate tests that are clinically unusable.

At the same time, African pharmacogenomics is supported by expanding research networks, biobanks, training programs, and regional collaborations [23]. Proposed strategies include regional reference laboratories, locally prioritized gene–drug pairs, workforce development, pharmacoeconomic evaluation, and phased clinical pilots [23,24]. In settings without mature electronic health records, patient-held safety cards, mobile tools, and structured paper reports may be more practical than centralized informatics. The Nigerian iPROTECTA model demonstrates how such tools can be incorporated into SCD care [15].

South African authors have proposed a phased strategy organized around research, implementation, and education [24]. Such a strategy could begin with high-priority gene–drug pairs, evaluate pharmacoeconomic impact, and expand only after local performance is established. For SCD, codeine and tramadol may offer a focused entry point because the clinical recommendations are clearer than for most other opioids.

Existing initiatives include Human Heredity and Health in Africa, regional genomic training programs, national genome projects, and the African Pharmacogenomics Consortium [23]. These efforts have strengthened capacity and data generation, although many remain dependent on external funding and have uneven links to routine care. Sustainable implementation will require national policy support, reimbursement pathways, local laboratory maintenance, and a workforce able to interpret results.

Pharmacoequity in SCD requires more than access to tests developed elsewhere. It requires African-led studies, locally valid phenotype translation, context-specific clinical pathways, and sustained capacity to update evidence as new alleles and structural variants are characterized. Without those elements, precision prescribing may widen existing disparities or deliver unreliable recommendations to the populations most affected by SCD.

Equity also requires attention to those who benefit from implementation. Programs should monitor whether testing is available across urban and rural sites, whether results are communicated in understandable formats, and whether recommended alternatives are accessible. A pharmacogenetic result has limited value if the suggested medication is unaffordable or unavailable. Clinical utility must therefore be evaluated in the context of the local formulary and referral environment.

## 7. Discussion

### 7.1. State of Readiness for Pharmacogenomics-Guided Opioid Prescribing

The evidence supports a staged interpretation of readiness. Targeted CYP2D6-informed prescribing is ready for selected decisions involving codeine and tramadol because the biological relationship is well established and formal guidance addresses poor, intermediate, normal, and ultrarapid metabolizers [5,8]. SCD-specific programs show that testing can be implemented in pediatric and African settings [14,15]. These findings justify testing when codeine or tramadol is being considered, and the result is not already available, particularly where those drugs remain common options.

“Ready” does not mean that every patient must be tested immediately. Testing may add little when a patient already has a well-documented, effective, and tolerated regimen, or when codeine and tramadol are not under consideration. It is more compelling when treatment history is limited, prior response is unexplained, or the health system can store the result for future use. Clinical context should therefore determine whether testing is reactive, preemptive, or unnecessary.

The evidence does not support universal pharmacogenetic testing before every opioid prescription. Hydrocodone is less strongly actionable, and routine CYP2D6-guided oxycodone prescribing is not supported [8,13]. Evidence for OPRM1, COMT, UGT2B7, and other candidate genes is not sufficiently consistent for routine opioid selection [3,4,5,6,8]. Preemptive multigene testing is conceptually attractive because SCD involves repeated medication exposure, but disease-specific outcome data remain limited [7,20,21,22].

Readiness should therefore be defined by the clinical decision. The most defensible current use is avoidance or selection of codeine and tramadol based on CYP2D6 phenotype. Broader panels and algorithms should remain within implementation studies or carefully governed clinical programs until they demonstrate patient benefit.

This staged position balances enthusiasm with evidentiary limits. It recognizes a clinically useful CYP2D6 signal without overstating the maturity of opioid pharmacogenomics as a whole. It also avoids a false choice between universal testing and no testing. The evidence supports targeted use now, structured evaluation of preemptive testing next, and continued research on less mature gene–drug relationships.

### 7.2. Limitations of Genotype-Only Prescribing

CYP2D6 genotype does not always equal the metabolic activity present at the time of prescribing. Phenoconversion occurs when medications or other clinical factors alter enzyme activity so that the functional phenotype differs from the genotype-predicted phenotype [25,26]. Strong CYP2D6 inhibitors such as paroxetine, fluoxetine, and bupropion can convert a predicted normal metabolizer into a functional intermediate or poor metabolizer. Moderate inhibitors can also reduce activity, particularly when several interacting drugs are used [25,26,27].

Estimates from CYP2D6-treated populations suggest that clinically relevant phenoconversion may affect a substantial proportion of patients receiving interacting drugs, although the magnitude varies by setting and definition [25]. In SCD, antidepressants and adjuvant analgesics may be prescribed alongside opioids, making inhibitor review particularly important. Medication reconciliation should include prescribed, over-the-counter, and recently discontinued agents because enzyme inhibition may not align neatly with a single encounter.

For codeine and tramadol, phenoconversion can reduce active-metabolite formation and cause treatment failure despite a genotype that predicts normal metabolism [25,26,27]. Polypharmacy increases this risk because multiple inhibitors may converge on the same pathway and because medication exposure changes across encounters. The relevant clinical question is therefore not only “What is the genotype?” but also “What is the likely enzyme activity under the current regimen?”

Renal and hepatic function further shape opioid response. SCD-associated kidney and liver injury may alter parent-drug and metabolite clearance, while reduced hepatic reserve may amplify the effects of enzyme inhibition [5,25,27]. These factors become more important with age and cumulative organ damage. A genotype-guided recommendation should therefore be paired with medication reconciliation and current renal and hepatic assessment.

Organ function can also alter the safety of alternative opioids selected after a CYP2D6 result. Avoiding codeine or tramadol does not automatically identify a safe substitute. Morphine, hydromorphone, methadone, and other options differ in renal handling, hepatic metabolism, half-life, and interaction profile. Pharmacogenetic guidance should therefore trigger a broader medication assessment rather than a simple one-for-one substitution.

Concomitant disease-modifying and supportive therapies may further confound observed opioid response, although SCD-specific evidence remains limited [5]. Hydroxyurea use, disease severity, and changes in care setting may be associated with both analgesic exposure and clinical outcomes, making causal interpretation difficult. Genotype should be treated as one component of a dynamic prescribing assessment rather than a permanent stand-alone answer.

These limitations do not negate the value of CYP2D6 testing. They define the conditions under which it should be used. A high-quality prescribing decision integrates stable genetic information with time-varying clinical information. Decision support should make that integration visible and should avoid presenting a genotype-derived phenotype as an immutable measure of current metabolism.

### 7.3. Pain Phenotype as an Independent Determinant of Response

Pharmacogenetics cannot determine whether the pain mechanism being treated is likely to respond to an opioid. SCD pain includes acute vaso-occlusive, chronic, and mixed states, and the same patient may move among them over time [1,2]. Nociceptive, neuropathic, and nociplastic mechanisms may overlap. A favorable CYP2D6 phenotype may therefore coexist with pain that is only partly opioid-responsive, while a poor-metabolizer phenotype may identify a drug failure in otherwise opioid-responsive nociceptive pain.

The Assessment of Pain through Patient-Reported Outcomes and other multidimensional frameworks can support this distinction, but no single instrument captures all relevant dimensions. Clinicians may need separate measures for pain intensity, temporal pattern, neuropathic features, function, mood, sleep, and social participation. The purpose is not to create a burdensome battery, but to identify the mechanism and impact most likely to change treatment.

Pain subphenotyping can be organized by chronicity, mechanism, variability, and functional impact. In a secondary analysis of the Pain in Sickle Cell Epidemiology Study, pain intensity, frequency, and day-to-day variability identified clinically distinct clusters with different opioid use, physical function, coping, and healthcare utilization [28]. Pain variability added information beyond average intensity and may help identify patients with unstable or persistent high-burden trajectories.

These patterns may influence how pharmacogenetic results are interpreted. Recurrent medication use in a high-variability phenotype may reflect fluctuating disease activity rather than metabolic failure, while persistent poor response despite an appropriate phenotype may suggest a different pain mechanism or substantial psychosocial amplification. Longitudinal pain data are therefore more informative than a single pain score when evaluating opioid response.

Mechanistic assessment is also important. In a study of individuals with HbSS, painDETECT screening suggested that neuropathic features were common [29]. Neuropathic pain reflects injury or dysfunction of the somatosensory system, while nociplastic pain reflects altered nociception and central sensitization. Both may require adjuvant or multimodal treatment rather than opioid escalation alone [4,29].

Screening tools cannot by themselves establish a mechanism, but they can identify patients who need further assessment. For example, neuropathic features may support consideration of non-opioid adjuvants, while nociplastic features may increase the value of behavioral, rehabilitative, sleep, and self-management interventions. Pharmacogenetics is most useful when embedded in this mechanism-based treatment plan.

Functional impact provides another clinically meaningful dimension. The Sickle Pain-Related Impact study distinguished high-impact chronic pain from lower-burden chronic pain according to restriction of work and other life activities [30]. High-impact chronic pain was associated with worse sleep, emotional well-being, social participation, and daily medication use. Pain status also changed over time, supporting repeated reassessment rather than a fixed classification [30].

These findings place pharmacogenomics within a broader precision-pain framework. CYP2D6 may explain why codeine or tramadol fails pharmacologically, but pain phenotype helps determine whether an opioid is the appropriate mechanistic choice. Validated measures of pain impact, neuropathic features, catastrophizing, sleep, and participation can help distinguish the need for a different opioid from the need for adjuvant, behavioral, rehabilitative, or multidisciplinary treatment [28,29,30].

This integrated model also helps prevent inappropriate opioid escalation. A genotype that predicts normal CYP2D6 activity should not be interpreted as evidence that a higher dose is required when pain remains uncontrolled. Conversely, a poor-metabolizer result should not be used to dismiss the patient’s pain. It should prompt selection of a more appropriate medication and reassessment of the pain phenotype.

### 7.4. Clinical and Implementation Implications

Implementation requires more than ordering a test. Results must be translated into a phenotype, adjusted for interacting drugs, and linked to a specific medication decision. Electronic health record alerts, pharmacist interpretation, structured reports, and patient-held safety tools have each been used to support this process [10,14,15]. Qualitative work in pediatric SCD also shows that clinicians need concise recommendations and better visibility of prior analgesic response [31].

Clinical reports should answer four questions: what phenotype was predicted, which medications are affected, what action is recommended, and what factors could modify the recommendation. Reports should also document assay limitations and the date of interpretation. Because genotype-to-phenotype standards can change, health systems need a process for reinterpretation and patient notification.

Decision support should distinguish strong from weak evidence. Alerts are most defensible for codeine and tramadol in poor or ultrarapid metabolizers. Less definitive findings should not generate the same level of interruption. Over-alerting may reduce clinician trust, while vague reports may fail to change prescribing. Systems should also prompt review of CYP2D6 inhibitors, renal and hepatic function, and pain phenotype.

Education should be role-specific. Prescribers need to understand when to order testing and how to choose alternatives; pharmacists can assess interactions and phenotype translation; nurses and pain teams can reinforce patient communication; and patients need a clear explanation that the result predicts drug handling rather than pain legitimacy. These functions are central to safe implementation.

Equitable implementation requires different delivery models across settings. High-resource systems may rely on integrated electronic records and clinical pharmacogenetics services. Other settings may use medication safety cards, mobile tools, or regional laboratories [15,23,24]. Sustainability, reimbursement, workforce education, patient understanding, and data governance should be evaluated alongside clinical outcomes.

## 8. Future Directions

Future research should first test CYP2D6-guided opioid care prospectively in SCD. Trials should compare genotype-informed selection with usual care and should distinguish codeine and tramadol from less actionable opioids. Important outcomes include time to adequate analgesia, treatment failure, opioid switching or escalation, adverse effects, emergency visits, hospitalization, and cumulative opioid exposure [5,11,12,13,15].

Trials should prespecify how genotype results alter prescribing and should document adherence to the recommendation. Without a clear treatment algorithm, a negative trial may reflect implementation failure rather than lack of biological utility. Stratification by age, care setting, baseline opioid exposure, and pain phenotype will help identify where benefit is greatest.

Second, studies should use longitudinal outcomes that matter to patients. Pain intensity alone does not capture function, sleep, participation, neuropathic features, or high-impact chronic pain [28,29,30]. Pharmacogenetic outcomes should therefore be paired with validated pain and quality-of-life measures. This will clarify whether a prescribing change improves the broader pain trajectory rather than only a laboratory or pharmacokinetic endpoint.

Patient-reported outcomes should be collected repeatedly because pain and function change over time. Studies should also measure trust, treatment satisfaction, and perceived access to analgesia. These outcomes are important in SCD, where poorly implemented precision tools could be experienced as another barrier to pain treatment.

Third, investigators should develop integrated clinicogenomic algorithms. These models should combine CYP2D6 phenotype with current CYP2D6 inhibitors, renal and hepatic function, age, medication burden, and pain phenotype [25,26,27,28,29,30]. Prospective validation is needed to determine whether such models outperform genotype-only recommendations and remain interpretable at the point of care.

Algorithms should remain transparent enough for clinicians to understand why a recommendation was generated. External validation across institutions and ancestry groups will be essential. Models that perform well only within one laboratory or electronic record system will have limited generalizability.

Fourth, implementation studies should evaluate feasibility, cost, sustainability, and equity. Research should compare electronic decision support, pharmacist-led services, patient-held safety cards, and regional laboratory models across different care settings [10,14,15,20,21,22,23,24]. Outcomes should include clinician adherence, patient understanding, turnaround time, alert burden, maintenance of results across institutions, and cost-effectiveness.

Economic evaluation should account for test cost, repeat use across the lifespan, avoided adverse events, reduced ineffective prescribing, and the resources required for interpretation and decision support. Cost-effectiveness may differ substantially between a reactive single-gene test, a preemptive panel, and a population-level program.

Fifth, larger African and African-ancestry cohorts are needed to characterize reduced-function alleles, copy-number variation, and haplotypes that are poorly represented in current reference datasets [15,16,17,18,19,22,23,24]. Local phenotype translation and treatment pathways should be validated rather than assumed to generalize from European-ancestry populations.

Research partnerships should prioritize African leadership, shared governance, local analytic capacity, and return of clinically useful results. Building reference datasets without strengthening implementation capacity would not address the translational gap. Harmonized methods can support comparison across countries while preserving locally relevant panel design.

Finally, the value of preemptive testing across the SCD lifespan should be tested directly. Studies should determine whether early multigene testing improves not only opioid selection but also the safety and efficiency of supportive-care prescribing over time [7,20,21,22]. Until those outcomes are demonstrated, preemptive panels should be viewed as promising infrastructure rather than established standard care.

A pragmatic development pathway would begin with clearly actionable CYP2D6-opioid pairs, build infrastructure for result storage and interaction review, and then expand to additional genes only as evidence and local clinical utility justify inclusion. This sequence would align implementation burden with the strength of the evidence.

## 9. Conclusions

CYP2D6-guided selection or avoidance of codeine and tramadol represents the clearest current application of opioid pharmacogenetics in SCD. However, the evidence does not support universal pharmacogenetic testing before all opioid prescriptions or a genotype-only approach to care. Clinically meaningful precision prescribing requires integration of pharmacogenomic results with pain phenotype, medication interactions, prior treatment response, and renal and hepatic function. Hydrocodone is less clearly actionable, and evidence remains insufficient for routine CYP2D6-guided oxycodone prescribing. Prospective SCD-specific studies are needed to determine whether targeted or preemptive testing improves analgesia, reduces toxicity and acute-care utilization, and can be implemented sustainably and equitably in the populations most affected by SCD.

## Figures and Tables

**Table 1:** CYP2D6 phenotype and implications for codeine and tramadol.

CYP2D6 Phenotype	Activity Score	Expected Effect	Clinical Implication
Ultrarapid metabolizer	>2.25	Increased active-metabolite formation; toxicity risk	Avoid codeine and tramadol
Normal metabolizer	1.25–2.25	Expected active-metabolite formation	Use standard recommended dosing
Intermediate metabolizer	0.25–1.0	Reduced active- metabolite formation; possible inadequate analgesia	Use a standard starting dose with close monitoring; consider an alternative if ineffective
Poor metabolizer	0	Little or no active- metabolite formation; likely treatment failure	Avoid codeine and tramadol

Note: Activity-score categories and recommendations are based on the CPIC framework [8]. Evidence is less definitive for hydrocodone and insufficient for routine CYP2D6-guided oxycodone prescribing.

## Data Availability

No new data were created or analyzed in this study.

## Abbreviations

The following abbreviations are used in this manuscript:

5-HT3	5-hydroxytryptamine receptor 3
AAPT	ACTTION–American Pain Society Pain Taxonomy
ABCB1	ATP-binding cassette subfamily B member 1
AFCAD	African Collaborative Initiative to Advance Diagnostics
AGMT	African Genomic Medicine Training Initiative
APC	African Pharmacogenomics Consortium
APOL1	Apolipoprotein L1
ASCQ-Me	Adult Sickle Cell Quality of Life Measurement Information System
CLIPMERGE	Clinical Implementation of Personalized Medicine through Electronic Health Records and Genomics
COMT	Catechol-O-methyltransferase
CPIC	Clinical Pharmacogenetics Implementation Consortium
CYP2B6	Cytochrome P450 2B6
CYP2C19	Cytochrome P450 2C19
CYP2C9	Cytochrome P450 2C9
CYP2D6	Cytochrome P450 2D6
H3Africa	Human Heredity and Health in Africa
HbSS	Hemoglobin SS
iPROTECTA	Implementing Pharmacogenomics Testing for Effective Care and Treatment in Africa
OPRM1	Opioid receptor mu 1
PREPARE	PREemptive Pharmacogenomic Testing for Preventing Adverse Drug Reactions
PROMIS	Patient-Reported Outcomes Measurement Information System
SAHGP	Southern African Human Genome Programme
SCD	Sickle cell disease
UGT2B7	UDP-glucuronosyltransferase 2B7

## References

[1] Smith W.R., Valrie C.R., Jaja C., Kenney M.O. (2023). Precision, integrative medicine for pain management in sickle cell disease. Front. Pain Res..

[2] Sil S., Manikowski A., Schneider M., Cohen L.L., Dampier C. (2022). Identifying chronic pain subgroups in pediatric sickle cell disease: A cluster-analytic approach. Clin. J. Pain.

[3] Di Salvo C., Valdiserra G., Balestrieri S., Beucci G., Paciulli G., Luculli G.I., De Vita A., Fornai M., Di Paolo A., Antonioli L. (2026). The role of clinical pharmacogenetics and opioid interactions in pain management: Current evidence and future perspectives. Pharmaceutics.

[4] Scott E.N., Simonson L.P., Loucks C.M. (2026). Personalized pain management: The use of pharmacogenomics in pain treatment strategies. Can. J. Physiol. Pharmacol..

[5] Elshaikh R.H., Babker A.M., Hussein S.E., Ahmed K.A.M., Sah A.K., Alfeel A.H. (2026). Pharmacogenomics and opioid efficacy in sickle cell disease. Medicina.

[6] Martin da Silva I., Plaza-Díaz A., Ruiz-Ramos J., Juanes-Borrego A., Riera P. (2025). The role of pharmacogenetic biomarkers in pain. Biomedicines.

[7] Gallaway K.A., Sakon C., Ongeri J., Patel K.S., Oliver J., Patacca H., O’Brien A.R.W., Skaar T.C., Tillman E.M. (2022). Opportunity for pharmacogenetics testing in patients with sickle cell anemia. Pharmacogenomics.

[8] Crews K.R., Monte A.A., Huddart R., Caudle K.E., Kharasch E.D., Gaedigk A., Dunnenberger H.M., Leeder J.S., Callaghan J.T., Samer C.F. (2021). Clinical Pharmacogenetics Implementation Consortium guideline for CYP2D6, OPRM1, and COMT genotypes and select opioid therapy. Clin. Pharmacol. Ther..

[9] Cho Y., Karrison T., Jack M.M., Choksi A.R., Knoebel R.W., Yeo K.-T.J., Volchenboum S.L., Szmulewitz R.Z., Vokes E.E., Ratain M.J. (2024). Catalyzing pharmacogenomic analysis for informing pain treatment (C-PAIN): A randomized trial of preemptive CYP2D6 genotyping in cancer palliative care. J. Pain Res..

[10] Ramsey L.B., Prows C.A., Chidambaran V., Sadhasivam S., Quinn C.T., Teusink-Cross A., Tang Girdwood S., Dawson D.B., Vinks A.A., Glauser T.A. (2023). Implementation of CYP2D6-guided opioid therapy at Cincinnati Children’s Hospital Medical Center. Am. J. Health Syst. Pharm..

[11] Reizine N., Danahey K., Schierer E., Liu P., Middlestadt M., Ludwig J., Truong T.M., van Wijk X.M.R., Yeo K.-T.J., Malec M. (2021). Impact of CYP2D6 pharmacogenomic status on pain control among opioid-treated oncology patients. Oncologist.

[12] Cavallari L.H., Myers R.A., Chakraborty H., Skaar T.C., Gray C.F., Baye J.F., Volpi S., Rider R., Cicali E.J., Elwood E.N. (2026). CYP2D6-guided opioid management and postoperative pain control: A randomized clinical trial. JAMA Netw. Open.

[13] Lteif C., Myers R.A., Elwood E.N., Harris E.C., Chakraborty H., Dexter P.R., Peterson J.F., Rider R., Skaar T.C., Volpi S. (2026). CYP2D6 phenotype and post-surgical pain control with hydrocodone and oxycodone. Front. Pharmacol..

[14] Gammal R.S., Crews K.R., Haidar C.E., Hoffman J.M., Baker D.K., Barker P.J., Estepp J.H., Pei D., Broeckel U., Wang W. (2016). Pharmacogenetics for safe codeine use in sickle cell disease. Pediatrics.

[15] Adeagbo B., Olarewaju O., Orherhe O., Chikwambi Z., Mazhindu A., Bolarinwa R., Bolaji O., Masimirembwa C., Consortium for Genomics and Therapeutics in Africa (2025). CYP2D6 pharmacogenetics in Nigerian sickle cell disease: Phase 1 of implementing pharmacogenomics testing for effective care and treatment in Africa (iPROTECTA) program. Gates Open Res..

[16] Yee M.M., Josephson C., Hill C.E., Harrington R., Castillejo M.-I., Ramjit R., Osunkwo I. (2013). Cytochrome P450 2D6 polymorphisms and predicted opioid metabolism in African-American children with sickle cell disease. J. Pediatr. Hematol. Oncol..

[17] Mapira N.L., Thelingwani R.S., Chikwambi Z., Kuona P., Masimirembwa C. (2023). Pharmacogenetics of pain management in Zimbabwean patients with sickle cell disease. Pharmacogenomics.

[18] Thomford N.E., Abraham S.A., Nyarko S.B., Biney R.P. (2024). A consideration of CYP2D6 genetic variations in the Ghanaian population as a potential ‘culprit’ for the tramadol ‘abuse crisis’. BMC Med. Genom..

[19] Nkya S., Makani J., Flanagan J.M. (2026). Genetics and genomics in sickle cell disease in Africa. Am. J. Hematol..

[20] Niedrig D.F., Rahmany A., Heib K., Hatz K.-D., Ludin K., Burden A.M., Béchir M., Serra A., Russmann S. (2021). Clinical relevance of a 16-gene pharmacogenetic panel test for medication management in a cohort of 135 patients. J. Clin. Med..

[21] Peruzzi E., Roncato R., De Mattia E., Bignucolo A., Swen J.J., Guchelaar H.-J., Toffoli G., Cecchin E. (2025). Implementation of pre-emptive testing of a pharmacogenomic panel in clinical practice: Where do we stand?. Br. J. Clin. Pharmacol..

[22] Hurrell T., Naidoo J., Masimirembwa C., Scholefield J. (2024). The case for pre-emptive pharmacogenetic screening in South Africa. J. Pers. Med..

[23] Mukabyiringiro E., Kinyanjui A.W., Nthenya G.R., Ndiege O.Z., Majok D.A., Inshutiyimana S., Terefe E.M. (2026). Strengthening pharmacogenomics implementation in Africa: Challenges and opportunities—A narrative review. Health Sci. Rep..

[24] Keuler N., Leuschner M., Hurrell T., Coetzee R., Jordaan B., Breedt W., Schellack N. (2026). A call to action for pharmacogenomics stewardship in South Africa. Front. Pharmacol..

[25] Nahid N.A., Johnson J.A. (2022). CYP2D6 pharmacogenetics and phenoconversion in personalized medicine. Expert Opin. Drug Metab. Toxicol..

[26] Cicali E.J., Wiisanen K. (2022). The importance of phenoconversion when using the CYP2D6 genotype in clinical practice. Pharmacogenomics.

[27] Stingl J.C., Viviani R. (2025). Pharmacogenetic guided drug therapy—How to deal with phenoconversion in polypharmacy. Expert Opin. Drug Metab. Toxicol..

[28] Bakshi N., Sinha C.B., Ross D., Krishnamurti L., Brandow A.M., Dampier C., DeBaun M.R., Yawn B.P., Shah N., Kanter J. (2022). Pain phenotypes in sickle cell disease and the role of pain variability. Pain.

[29] Mohan M., Swathi V., Aggarwal P., Rathnamma S., Bhat D. (2026). Evaluation of the neuropathic component of pain in sickle cell disease. Hemoglobin.

[30] Jagtiani A., Hawk A., Sinha C., Gillespie S.E., Mirbeyk M., Burke R., Ifendu N., Reese S., Howard V., Boguslawski S. (2026). A prospective study of high-impact chronic pain in sickle cell disease: The Sickle Pain-Related Impact (SPiRIt) study. Pain.

[31] Rosenblatt A., Williams V., Lamb N., Manworren R. (2026). Knowledge translation of pharmacogenomics for pain management in patients with sickle cell disease: A qualitative study. J. Pediatr. Hematol. Oncol..

